# Time to Move From Mandatory Stretching? We Need to Differentiate “Can I?” From “Do I Have To?”

**DOI:** 10.3389/fphys.2021.714166

**Published:** 2021-07-22

**Authors:** José Afonso, Jesús Olivares-Jabalera, Renato Andrade

**Affiliations:** ^1^Faculty of Sport of the University of Porto (FADEUP), Centre for Research, Education, Innovation and Intervention in Sport (CIFI_2_D), Porto, Portugal; ^2^Sport Research Lab, Football Science Institute, Granada, Spain; ^3^Department of Physical and Sports Education, Sport and Health University Research Institute (iMUDS), University of Granada, Granada, Spain; ^4^Clínica do Dragão, Espregueira-Mendes Sports Centre – FIFA Medical Centre of Excellence, Porto, Portugal; ^5^Dom Henrique Research Centre, Porto, Portugal; ^6^Porto Biomechanics Laboratory (LABIOMEP), Faculty of Sport, University of Porto, Porto, Portugal

**Keywords:** stretching, exercise prescription, injury prevention, warm-up, cool-down, range of motion

## Introduction

Flexibility is the ability to move through full joint range of motion (ROM), while stretching is an intervention to improve flexibility and achieve other goals (e.g., post-exercise relaxation) (ACSM, 2021). Stretching has been promoted as mandatory in exercise programs (Behm, 2019; American Heart Association, 2020; ACSM, 2021), although this is changing toward an optional feature (Bull et al., 2020). There are different types of stretching, including active static stretching (SS—active lengthening of a muscle until the feeling of stretch or to the point of discomfort), passive static stretching (PS—where an external force is applied, e.g., by a coach or a colleague), dynamic stretching (DS –controlled movements through the joint ROM) and proprioceptive neuromuscular facilitation (PNF—combining PS with isometric contractions) (Behm, 2019). We will focus on SS and PS, since these methods are at the heart of most debates, with the pendulum swinging across the years (Behm et al., 2021b). The answer to “Can I perform a given exercise intervention?” is straightforward: when the benefits of an intervention outweigh its adverse effects or contra-indications, the answer is “yes.” Let us take the example of a study with 15 University students (Bengtsson et al., 2018), to illustrate the difference between the two questions: the negative acute effects of SS during a warm-up were restored if followed by isokinetic contractions, suggesting that SS can be included in a comprehensive warm-up protocol. University students are not representative of athletes, and a small sample does not warrant generalizations, but our point is that answering the first question (“Can I?”) does not answer the second question (“Do I have to?”). Focusing the research question and applicability on “Can I?” may be short-sighted. To date, we feel that research has focused more strongly on answering what stretching can do, while more information is required as to how stretching compares to alternative interventions. We will explore the differences between “Can I?” and “Do I have to?” stretch and their implications to warm-up, cool-down, ROM, and injury risk.

## “Can I” Vs. “Do I Have” To Stretch in the Warm-Up?

Arguments for including stretching during the warm-up comprise acute improvements in ROM (Behm et al., 2016), improved proprioception (Walsh, 2017), psychological preparedness (Blazevich et al., 2018) and reduction of injury risk (Behm et al., 2021b), none of which is exclusive to stretching (Blazevich and Babault, 2019; Prieske et al., 2020). The evidence suggests that SS and PS may impair acute performance in power-, strength-, and speed-related activities, but these effects are minor and can be mitigated if SS lasts 30 to 60 s per muscle group and is followed by dynamic warm-up activities (Behm and Chaouachi, 2011; Kay and Blazevich, 2012; Behm et al., 2016, 2021b). But if multiple, <60 s stretches are compounded, what are their accumulated effects? And how does stretching intensity affect the outcomes? Performing unilateral PS induces improvements in passive ROM in non-local and non-stretched joints (Behm et al., 2021a), and consecutive sets of SS provoke increases of parasympathetic activity that remain for 5 min post-SS (Farinatti et al., 2011; Inami et al., 2014). Further research is warranted.

A comprehensive warm-up including a pre-stretching warm-up, followed by SS/PS, and then followed by dynamic exercises (Reid et al., 2018; Behm et al., 2021b) demands a longer than necessary warm-up duration. Alternative warm-up protocols focusing on post-activation potentiation (PAP) or performance enhancement (PAPE) are based on high-intensity, dynamic actions (Blazevich and Babault, 2019; Prieske et al., 2020), dismissing the need for SS/PS. Another argument for including SS/PS in the warm-up concerns the *acute* improvements in ROM, but these tend to last up to 30 min (Behm et al., 2016), while training sessions last longer, especially when considering athletes (not so much in the recreational- or health-related contexts), for which repeated bouts of SS/PS during the training session would be required. Interventions such as DS (Behm et al., 2016), foam rolling (Wilke et al., 2020) and high-intensity resistance training (Moreno-Pérez et al., 2021) also acutely improve ROM. The issue of *chronic* improvements in ROM is more complex and will be addressed in a separate section. For now, the answer to “Can I stretch in the warm-up?” is “probably yes”; but to the question “Do I have to stretch in the warm-up?” the answer is “maybe not.”

## “Can I” Vs. “Do I Have” To Stretch in the Cool-Down Phase?

Post-exercise stretching is prescribed under the belief that it enhances recovery (American Heart Association, 2020; ACSM, 2021), but reviews do not support these claims (Herbert and Gabriel, 2002; Henschke and Lin, 2011; Herbert et al., 2011; Torres et al., 2012; Baxter et al., 2017; Van Hooren and Peake, 2018). A systematic review with meta-analysis including 11 randomized controlled trials (RCTs) assessed the effects of post-exercise stretching on short-term (≤ 1 h after exercise) and delayed (24, 48, 72 h) recovery markers including delayed onset muscular soreness (DOMS), strength and ROM (Afonso et al., 2021a). Comparing with passive recovery (i.e., rest) or other recovery methods (e.g., low-intensity cycling), SS and PS showed no additional benefits in any of the outcomes, at any time point, in contrast with existing guidelines (American Heart Association, 2020; ACSM, 2021).

The argument that some athletes feel better when stretching (Judge et al., 2020) is misleading, as the subjective sensation of feeling better may not translate into objective measurable improvements (Afonso et al., 2021a). And it is a dangerous argument, as it could easily be looked from the other side, whereby athletes that do not like to stretch would be evidence against stretching—so, we feel that these lines of argumentation should be avoided. The argument that stretching provides team bonding (Behm et al., 2021b) also misses the mark: other activities can promote team bonding. In sum: “Can I stretch in the cool-down?” Probably yes, but when performed, post-exercise stretching should eschew high intensities (Behm, 2019). “Do I have to stretch in the cool-down?” Probably not, as evidence suggests it does not enhance recovery.

## “Can I” Vs. “Do I Have To” Stretch To Chronically Improve Range of Motion?

Stretching can chronically improve ROM (Blazevich, 2018) by increasing fascicle length, improving stretch tolerance, altering pennation angles and reducing tonic reflex activity (Guissard and Duchateau, 2004; Blazevich et al., 2014), but it is not the only method capable of doing so (Saraiva et al., 2014; Afonso et al., 2021b). Stretching may be required for sports that demand extreme ROM, but for most sports and general population, there are alternative interventions available, such as resistance training (Saraiva et al., 2014; Nuzzo, 2020) and foam rolling exercises (Aune et al., 2019). A systematic review with meta-analysis of 11 RCTs comparing strength training to stretching for ROM gains has found that for interventions lasting 5 and 16 weeks, the strength training and stretching protocols did not differ in their effects on ROM (Afonso et al., 2021b). To different degrees, eccentric and concentric strength training with full ROM, as well as plyometric training, induce changes in muscle fascicle length and pennation angle, and tendon extensibility, resulting in ROM gains (Reeves et al., 2009; Kubo et al., 2017; Valamatos et al., 2018; Gérard et al., 2020; Marušič et al., 2020). However, the studies included in the review of Afonso et al. (2021b) had considerable heterogeneity in design, populations and protocols. The answer to the question “Can I stretch to improve ROM?” is Yes. But do I have to stretch to improve ROM? Possibly not, but more comparative research is required, and sports requiring extreme ROM should be considered separately.

## “Can I” Vs. “Do I Have To” Stretch To Reduce Injury Risk?

Several exercise types and sports require wide use of stretch-shortening cycles (Witvrouw et al., 2004), with muscles alternating between shortening and stretching phases. It has been proposed that stretching could thus reduce injury risk (Behm et al., 2021b), but this is contentious (Witvrouw et al., 2004; Nuzzo, 2020). The systematic review of Behm et al. (2016) showed no clear effect of SS or PNF stretching on all-cause or overuse injuries, and there was insufficient data available for DS. Other systematic reviews have failed to find an association between stretching and injury risk (Herbert and Gabriel, 2002; Weldon and Hill, 2003; Thacker et al., 2004; Small et al., 2008; Lauersen et al., 2014; Leppänen et al., 2014; Lewis, 2014; Dijksma et al., 2020). Based on one of the largest RCT with military individuals (Pope et al., 2000), a total of 337 individuals would need to undergo a stretching intervention to prevent a single lower-limb injury. The argument that stretching does not reduce overall risk of injury but might reduce risk of musculoskeletal injury (Reid et al., 2018) should consider a trade-off: if overall risk is the same but risk of musculoskeletal injury is reduced, other risks were possibly aggravated. Since injury mechanisms are usually multifactorial, isolating one factor (e.g., stretching) is difficult, and so definitive claims that stretching reduces (or not) injury risk should be avoided.

The link between flexibility and injury risk is unclear (Green et al., 2020). Some evidence shows association between flexibility and injury risk (de la Motte et al., 2019), but this should not be misinterpreted as a causal effect. There is evidence that an injury impairs ROM (Maniar et al., 2016), but the inverse may not be true—although it is reasonable to assume that ROM levels that are insufficient for the movement demands of a certain exercise or sport may expose the athlete to increased injury risk. Establishing a causal relationship between insufficient flexibility and increased injury risk would not support the mandatory utilization of stretching, as there are alternative interventions to improve flexibility, including strength training (Saraiva et al., 2014; Nuzzo, 2020; Afonso et al., 2021b), but more long-term comparative studies are required. The relationships between flexibility and technique also warrant more research. For now, the relationship between stretching and injury risk remains controversial (Green et al., 2020). The answer to “can I stretch?” is yes—it probably will not increase injury risk. But the answer to “do I have to stretch?” is “possibly no”—as the likelihood of decreasing the injury risk is contentious.

## Discussion

Establishing that stretching *can* be performed in different contexts is not the same as establishing that stretching *must* be performed. (1) SS and PS can be performed during the warm-up, especially if using durations <30 to 60 s per muscle group, but at the cost of longer warm-up routines. Alternative warm-up protocols do not require SS or PS, seem equally effective and require less time to implement. (2) SS and PS can be performed during the cool-down, but there are apparently no benefits in short-term or delayed recovery of strength and ROM and does not decrease the magnitude of DOMS. (3) SS and PS improve ROM if performed chronically, but so does strength training, which also has multiple health-related benefits (Bull et al., 2020). However, the scarcity of comparative studies and their heterogeneity advise against stronger conclusions. Sports requiring extreme ROM may require specific approaches. (4) There is no clear relationship between stretching and injury risk, which is partly expected as most injuries are multifactorial in nature. Associations between flexibility and injury risk do not necessarily support the utilization of stretching because: (i) association does not mean causation; and (ii) flexibility can be improved through other methods (although more long-term comparative research is needed). We should adopt nuanced approaches when recommending stretching. “Can I stretch?”: probably, yes. “Do I have to stretch?”: possibly, no. The answer may depend on the goals, population, timing of the year or season, and individual characteristics.

## Author Contributions

JA conceived the original draft. All authors had equally relevant contributions for writing and revising the manuscript.

## Conflict of Interest

The authors declare that the research was conducted in the absence of any commercial or financial relationships that could be construed as a potential conflict of interest.
